# Midgut malrotation with volvulus discovered at an emergency caesarean section for placental abruption

**DOI:** 10.4314/gmj.v55i1.14

**Published:** 2021-03

**Authors:** Kofi T Mensah, Raphael Kwarase, Stephenson Laari, William N A Thompson

**Affiliations:** Agogo Presbyterian Hospital, Box 27, Agogo, Ashanti-Akim, Ghana

**Keywords:** Midgut malrotation, volvulus, bowel, Ladd's bands, pregnancy

## Abstract

Adult midgut malrotation with volvulus (AMMV) is an uncommon presentation which may be found incidentally during abdominal radiologic investigations or at laparotomy. We report a case of AMMV and small bowel gangrene in a 35-year-old Gravida four, Para three at 39 weeks, 4 days gestation who presented with a short history of worsening abdominal pain, repeated vomiting and abdominal wall guarding. Emergency caesarean section performed on account of a suspected placental abruption incidentally revealed a long segment of non-viable small intestine. Subsequent midline laparotomy disclosed a midgut malrotation with volvulus and bowel gangrene. This resulted in a 4.6m resection of non-viable small bowel with Ladd's procedure. The patient developed moderate symptoms of short bowel syndrome in the post-operative period which was successfully managed non-operatively. This case report represents a rare diagnosis, in the West-African sub-region, of an adult midgut malrotation with volvulus mimicking a third trimester obstetric emergency.

**Funding:**

None declared

## Introduction

Intestinal malrotation is the incomplete or abnormal embryonic rotation of the midgut.^1^ It is a congenital condition, commonly diagnosed in the paediatric population with 64–80% of cases seen in the neonatal period and about 90% of cases diagnosed by the first year of life.^2^ The remainder of cases only get diagnosed incidentally, in the older child or adult at laparotomy or during radiologic investigations of the abdomen for other purposes.^3^ There is a reported 0.2% prevalence of adult midgut malrotation.^4^,^5^

Clinical presentation of adult midgut malrotation is more variable^6^ and may be chronic or acute. The acute clinical presentation, characterized by a variable combination of abdominal pain, vomiting, gastrointestinal bleeding and haemodynamic instability, has been reported to occur in only 10–15% of adult patients with the condition.^7^ The majority of adults with midgut malrotation are never diagnosed in their lifetime.^8^ We present a case of an adult midgut malrotation with volvulus and bowel gangrene presenting as an obstetric emergency.

## Case Report

A 35-year-old, Gravida 4 Para 3 at 39 weeks plus four days was referred to us with complaints of sudden onset of abdominal pains that morning. The pain, which had now become generalized, was marked in the epigastrum and radiated to the back. The pain became constant, and incapacitating associated with four episodes of bilious, non-bloody vomiting. There was no associated vaginal or rectal bleeding.

She had had three antenatal clinic visits starting at 19 weeks gestation. The first visit was necessitated by symptoms of intermittent abdominal pains and dizziness. She was treated for malaria after having a positive result for the malaria rapid diagnostic test. She had a single obstetric ultrasound scan (USG) at 25 weeks with normal findings. She had had no previous surgeries and no known comorbidities. Her blood pressure was 104/74 mmHg, pulse rate of 76 beats per minute (bpm) and a temperature of 36.6°C. The laboratory results were haemoglobin of 11.0gm/dl, a total white cell count of 10.33x10^9^/L, a platelet cunt of 294x10^9^/L and s negative sickling test.

The symphysis-fundal height was 35cm and the abdomen was moderately tense with generalized tenderness, guarding and rebound tenderness. Uterine contractions could not be palpated and the cervical os was closed.

Ultrasound scan showed a live singleton weighing 3.7kg with cephalic presentation, posterior fundal placentation, adequate liquor volume and a fetal heart rate of 155 beats per minute. A working diagnosis of placental abruption was made and after obtaining an informed consent, emergency caesarean section under spinal anaesthesia was performed.

**Figure 1:**
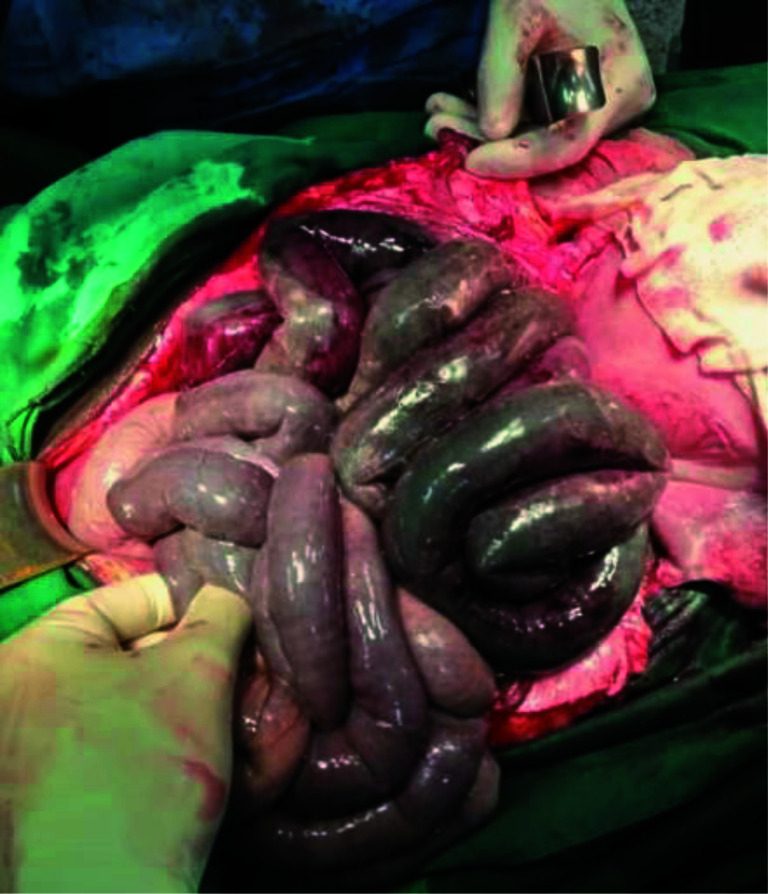
Initial appearance of bowel after midline exposure

**Figure 2:**
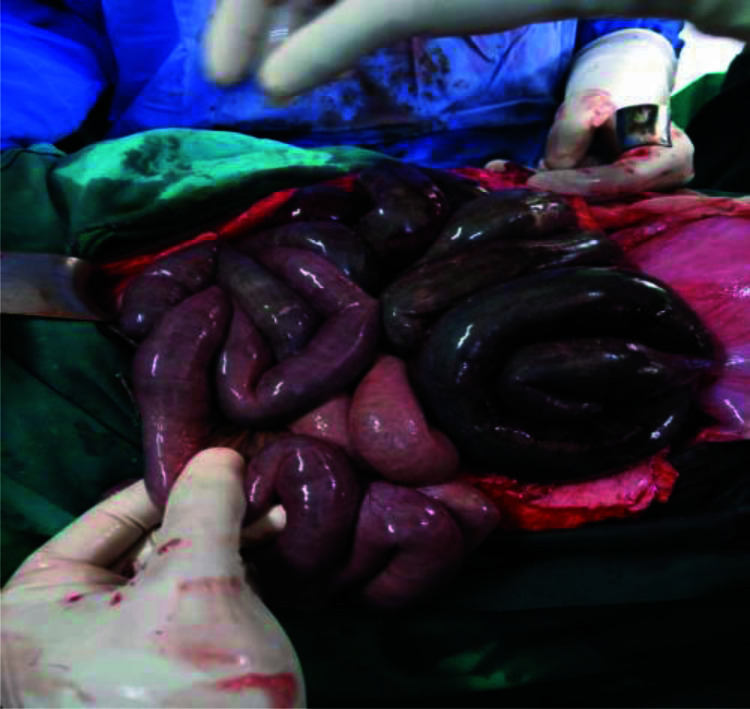
Portions of small bowel regaining some perfusion after de-torsion and division of strangulating bands

**Figure 3:**
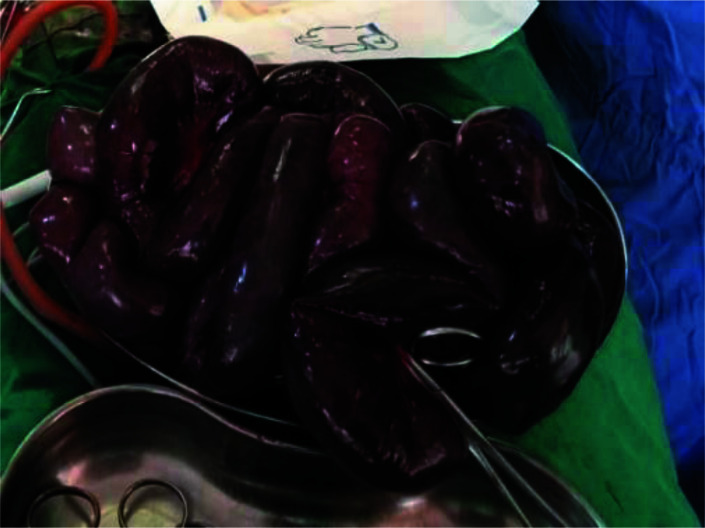
4.6m of non-viable small bowel resected

**Figure 4:**
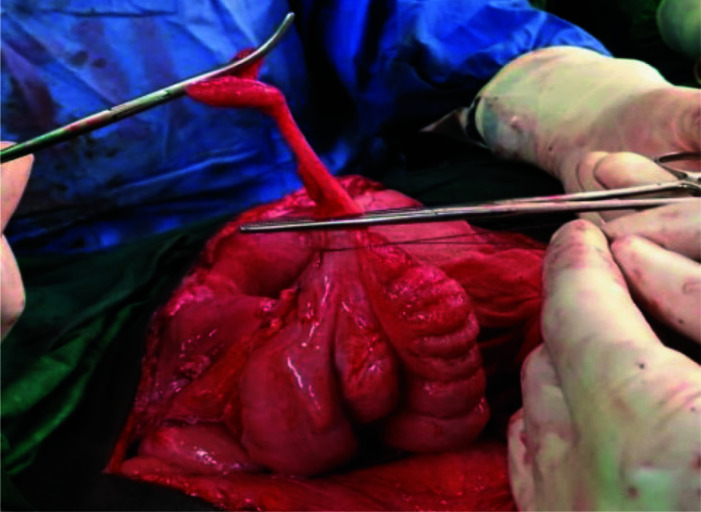
Appendicectomy step

**Figure 5:**
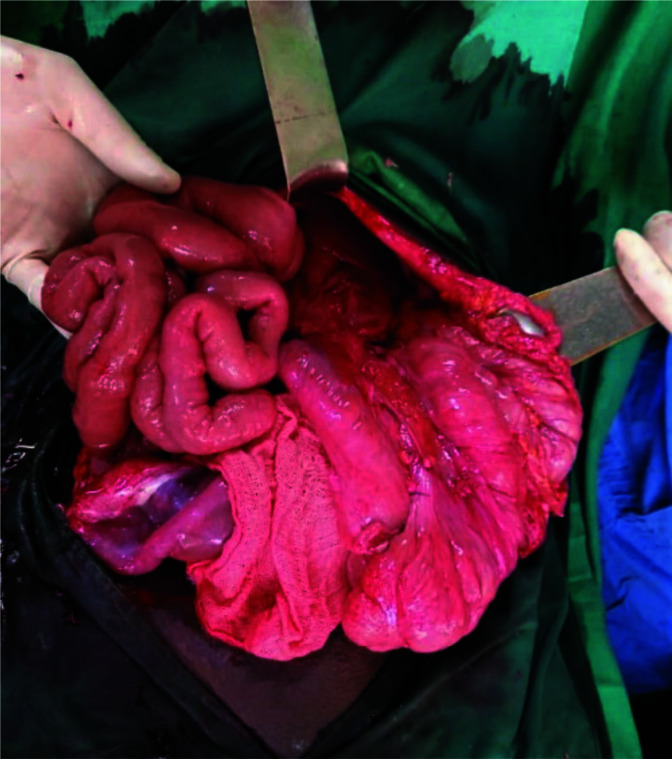
Replacement of the residual bowel the position of non-rotation (Small bowel on the right, large bowel on the left)

Intraoperatively, 600mls of serosanguinous peritoneal fluid was encountered. After delivering a healthy baby with APGARS 8 and 9, and repairing the uterus, it was noticed that the adjacent small bowel was greenish-black in appearance for which reason the surgical team was brought on board. After converting to general anaesthesia and with a midline incision, further exploration revealed a 4.6m length of jejuno-ileal gangrene from volvulus complicating a midgut malrotation (Figure 1). The proximal jejunum, distal ileum and entire large bowel were spared. The caecum was fixed to the right upper quadrant with Ladd's bands crossing medially and strangulating the root of the mesentery at the level of the duodenum. Counterclockwise detorsion of the small bowel was carried out. The constricting bands were ligated and divided followed by the application of warm packs to the bowel. This resulted in some improved bowel perfusion evidenced by a reddish-pink colour change to some 30cm of previously dusky-looking small intestine segments (Figure 2). Ultimately, 4.6m of non-viable small intestine had to be resected (Figure 3) with a single layer end-to-end anastomosis situated 13cm from the ileocaecal junction. The residual small intestine measured 85cm. This was followed by an appendicectomy (Figure 4) and positioning of the small bowel on the right side and the large bowel on the left (Figure 5).

Post-operative management was carried out in the high dependency unit by both the obstetric and surgical teams. Intravenous fluids and electrolyte replacements were administered along with broad spectrum antibiotics. Nasogastric decompression was discontinued after post-operative day (POD) 5 at which time normal peristalsis had resumed. Graduated oral intake along with anti-motility medications were also instituted. From POD 6, she started to pass, not less than six episodes per day, watery stools commensurate with short bowel syndrome necessitating an upward dose adjustment of the anti-motility drugs. In response, the stools became more solid and less than three episodes per day were recorded.

She was discharged on POD 13. She was reviewed twice in the first 30 days after discharge and was doing well. After missing subsequent review appointments, she returned with exacerbation of the short bowel syndrome. She was moderately dehydrated and had lost 9.3kg of weight.

She was readmitted for one week and adequately resuscitated before being discharged on oral medications, dietary adjustments and adherence counselling. She is doing well a year later with no new abdominal complaints.

## Discussion

The diagnosis of midgut malrotation is often delayed or missed leading to considerable morbidity and mortality.^9^,^10^,^11^ Midgut malrotation represents 1–% of all causes of intestinal obstruction in pregnancy.^12^ Most cases of midgut malrotation in pregnancy are diagnosed in the second and third trimesters and links have been established with the upward bowel displacement by the rapidly enlarging uterus^13^ and the effects of the hormone relaxin.^12^ To the best knowledge of the authors, no cases of adult midgut malrotation with volvulus have been reported incidentally at caesarean section in the West-African sub-region. The non-specific symptoms may be confused with labour pains in the pregnant patient.^14^ The colicky pain which transitioned to a constant abdominal pain in our patient should help clinicians distinguish a midgut volvulus from the intermittent pain of labour. We also suspect our patient's second trimester episode of intermittent abdominal pain and dizziness treated as malaria, to have been a subclinical volvulus occurring coincidentally with the malaria.

In a review of 23 cases of in-cyesis diagnosis of midgut malrotation over the last three decades, only 2 of the 23 patients did not require surgery and half of the third trimester diagnoses were made at surgery.^15^ Non-invasive imaging modalities include ultrasonography (USG), computed tomography (CT) scan and magnetic resonance imaging (MRI). The ultrasound scan performed for our patient at presentation did not comment on possible extra-uterine etiologies of the patient's abdominal pain such as gallstone disease, acute pancreatitis or acute appendicitis. Ultrasound detects a midgut malrotation when there is a reversal of the position of the superior mesenteric vein and the superior mesenteric artery.^16^ Ionizing radiation imaging modalities are, generally, not requested by clinicians in pregnancy although some second and third trimester diagnosis of adult midgut malrotation have been made with CT and MRI, both modalities being capable of picking up the whirlpool sign.^15^,^17^

Ultrasonography remains operator dependent and is not recommended for diagnosing midgut malrotation^7^ because a normal finding does not rule out the diagnosis.^18^ Notwithstanding, a skilled operator may detect the ultrasound 'whirlpool sign'^19^ The ‘whirlpool sign’ is a clockwise enfolding of the superior mesenteric vein and mesentery around the superior mesenteric artery.^20^

The typical manifestation of haemodynamic instability suggestive of bowel underperfusion from volvulus was absent in our patient despite having a 4.6m length of nonviable of small bowel. This could be due to the hyperdynamic pregnancy state masking the expected physiological derangements associated with bowel ischaemia.^15^ Clinicians must be aware of this possible presentation of adult midgut malrotation in cyesis and not delay resuscitation or surgical intervention. This reinforces the importance of operative care for virtually all cases of diagnosed midgut malrotation in pregnancy to forestall the consequences of volvulus to both the mother and baby.^3^ There have been reported cases of maternal and neoonatal mortalities due to delayed intervention^15^ or extensive bowel gangrene.^14^

Adult midgut malrotation with volvulus is usually not the first diagnosis a doctor would make for an acute abdominal pain in a multiparous woman at term. As such, the initial diagnosis of placental abruption made in this case is probably justified. It is possible that further delay in operative intervention could have resulted in more extensive bowel devascularization and a less favourable outcome. Time is key in all acute presentations of suspected placental abruption and thus, it was proper to proceed with immediate caesarean delivery, as opposed to the longer process of labour induction with a closed cervix. Additionally, the physiological stress of induced labour could have worsened the metabolic state of this patient. The patient would also have been less likely to cooperate with labour monitoring in the face of worsening peritonism limiting most forms of foeto-maternal maneuvres such as cardiotocography and rubbing up uterine contractions after delivery.

This risk from diagnostic delay highlights the importance of ensuring that medical doctors who perform caesarean sections are able to recognize such incidental pathologies and promptly consult or refer appropriately. This case presented, additionally, represents a call for efforts to ensure adequate surgical expertise in strategic district hospitals to handle such unexpected findings at caesarean delivery and avert the delays inherent in long-distance referrals to tertiary centres.

In the long-term, our patient remains at risk of recurrent volvulus (1.8 to 8%)^7^, small bowel adhesive obstruction (up to 15%)^21^ and relapsing complications of the severely shortened small bowel.^7^ Finally, the proposed role of the third trimester gravid uterus in erecting a static barrier that blocks the de-torsion of pre-existing intermittent volvulus in an adult midgut malrotation, thereby, precipitating an acute volvulus^15^, is worth investigating in future studies.

## Conclusion

Adult midgut malrotation with volvulus in pregnancy is rare and the clinician may never have a high enough index of clinical suspicion for its presence until it is diagnosed incidentally. However, where there are persistent non-specific abdominal symptoms in pregnancy which do not respond to conventional treatment, a heightened awareness from the clinician is required in order to make an early diagnosis of midgut malrotation. This would prevent the undesirable consequence of bowel gangrene from volvulus.
